# Psychophysical Evaluation of Achromatic and Chromatic Vision of Workers Chronically Exposed to Organic Solvents

**DOI:** 10.1155/2012/784390

**Published:** 2011-12-15

**Authors:** Eliza Maria da Costa Brito Lacerda, Monica Gomes Lima, Anderson Raiol Rodrigues, Cláudio Eduardo Correa Teixeira, Lauro José Barata de Lima, Dora Fix Ventura, Luiz Carlos de Lima Silveira

**Affiliations:** ^1^Instituto de Ciências Biológicas, Universidade Federal do Pará, 66075-110 Belém, PA, Brazil; ^2^Núcleo de Medicina Tropical, Universidade Federal do Pará, 66055-240 Belém, PA, Brazil; ^3^Instituto de Psicologia, Universidade de São Paulo, 05508-030 São Paulo, SP, Brazil; ^4^Núcleo de Neurociências e Comportamento, Universidade de São Paulo, 05508-030 São Paulo, SP, Brazil

## Abstract

The purpose of this paper was to evaluate achromatic and chromatic vision of workers chronically exposed to organic solvents through psychophysical methods. Thirty-one gas station workers (31.5 ± 8.4 years old) were evaluated. Psychophysical tests were achromatic tests (Snellen chart, spatial and temporal contrast sensitivity, and visual perimetry) and chromatic tests (Ishihara's test, color discrimination ellipses, and Farnsworth-Munsell 100 hue test—FM100). Spatial contrast sensitivities of exposed workers were lower than the control at spatial frequencies of 20 and 30 cpd whilst the temporal contrast sensitivity was preserved. Visual field losses were found in 10–30 degrees of eccentricity in the solvent exposed workers. The exposed workers group had higher error values of FM100 and wider color discrimination ellipses area compared to the controls. Workers occupationally exposed to organic solvents had abnormal visual functions, mainly color vision losses and visual field constriction.

## 1. Introduction

Studies about the effect of organic solvents in biological systems are more frequent in occupational medicine, and most commonly the intoxication is occupational and caused by solvent mixtures [1–7]. In addition, inhalation is the major pathway of intoxication in occupational environment [8–10].

 Occupational exposure to organic solvents can cause damage in both central and peripheral nervous system [11–14], and the visual system is one of the main targets of organic solvent intoxication [15]. As a result, acquired dyschromatopsias usually have been found in chronically exposed subjects to organic solvent mixtures [3, 16–19], as well as to specific solvents as n-hexane, styrene, and toluene [20–25]. Most color vision deficiencies due to exposure to solvents have subclinical symptoms, and a loss of the blue-yellow discrimination has been the most frequently reported impairment [3, 16, 18, 20–22, 24, 26–30], although some studies described altered red-green discrimination [3, 31].

 It has been described that chronic exposure to n-hexane may cause color discrimination losses, associated with maculopathy [32] and visual perimetry losses at the periphery, with optic nerve atrophy and retrobulbar neuritis [33]. Optic neuropathy is a finding associated with polyneuropathy in cases of alcohol, methanol, styrene, toluene, trichloroethylene, and solvent misture intoxication [34]. Decreased spatial contrast sensitivity in the middle range (6–12 cpd) of spatial frequencies associated to normal visual acuity seems to be an indicator of visual impairment induced by chronic exposure to styrene, acute exposure to tetrachloroethylene or triethylamine [21, 25, 35] and organic solvent mixtures [6, 36–38]. Losses of spatial vision can be dependent of the intoxication level [38–40].

Painters, factory workers, and cleaners are subject to continuous exposure to organic solvents. Investigation of their visual system to look for functional deficits has been performed by several authors, showing the impact of this exposure [6, 7, 25, 30, 41]. In some countries, Brazil included, automobile tanks are filled by gas station workers. Therefore, in this job the person is subject to a long period of organic solvent exposure. Automobile fuel is composed of a mixture of organic solvent including gasoline, alcohol, and diesel oil. They are composed by several hydrocarbons such as methane, ethane, propane, pentane, methanol, ethanol, propanol, methyl tertiary butyl ester, benzene, toluene, and xylene.

## 2. Methods

Thirty-one gas station workers agreed to participate in the study. Two subjects were excluded due to congenital red-green dyschromatopsia. Twenty-nine (27 males, 31.5 ± 8.4 years old) workers were evaluated. All procedures were evaluated by Ethical Committee in Research in Humans of the Tropical Medicine Nucleus of Federal University of Pará (Protocol no. 075/2006-CEP/NMT). These subjects had normal visual acuity or corrected to 20/20 (Snellen test).

Gas station workers participated in the current work according to their availability and in some cases they were unable to do all the tests due to their heavy work schedule. Control group for each psychophysical test was composed by the same number of subjects as the exposed group, matched in age and gender (32.8 ± 9.5 years old). The control subjects worked in environments free of solvent exposure.

### 2.1. Psychophysical Tests

Achromatic (spatial and temporal contrast sensitivity and visual perimetry) and chromatic (Farnsworth-Munsell 100 hue test, color discrimination ellipses) psychophysical tests were performed. Stimuli were displayed in a CRT high spatial and temporal resolution (Monitor Trinitron en Color Sony model CPG-G420). Spatial contrast sensitivity was measured using static vertical sinusoidal luminance gratings, of 6.5° × 5° of visual angle, and 43.5 cd/m^2^ mean luminance. Eleven spatial frequencies were used ranging between 0.2–30 cpd. Contrast thresholds were estimated using a staircase (10 reversals) protocol which started from subthreshold to suprathreshold contrasts. Contrast sensitivity was expressed as the inverse of contrast threshold values. Twenty-five workers were tested in spatial contrast sensitivity and the control group was composed by 25 subjects.

Temporal contrast sensitivity was measured using a square field (2.5° × 2.5° of visual angle) that flickered at seven temporal frequencies ranging between 0.5–32 Hz. The background luminance was equal to the mean stimulus luminance (43.5 cd/m^2^). A staircase procedure, analogous to that described for the spatial contrast sensitivity measurements, was used. Twenty-five workers were tested in temporal contrast sensitivity and the control group was composed by 25 subjects.

Visual perimetry assessment was performed using the Humphrey field analyzer (model 745, Humphrey System, CA). Central 10-2 (SITA-fast strategy, Central 30-2 (SITA-standard strategy) and Peripheral 60-4 (SITA-standard strategy) protocols were used. At each point in the visual field, thresholds were estimated using a staircase procedure, in which, correct responses were followed by a 4 dB luminance decrease, and mistakes by a 2 dB luminance increase. Results of visual perimetry were analyzed in eight eccentricity rings (0°–3.3°, 3.3°–6.6°, 6.6°–10°, 10°–20°, 20°–30°). Twenty-one workers were tested in visual perimetry and the control group was composed by 21 subjects.

Color discrimination was estimated by two different procedures: the Farnsworth-Munsell 100 hue (FM100) arrangement test and the Mollon-Reffin color test.

The FM 100 test consisted of 85 stimuli (each stimuli was a disk of 1° of visual angle, mean luminance of 41.75 cd/m^2^) of different hues and same saturation (30%), distributed in a chromatic axis in Munsell color space. At the beginning of the test, the subject was shown the correct sequence of the stimuli, arranged in a gradually changing order in the hue dimension in the Munsell color space. The stimuli were then disarranged and the subject was instructed to order the stimuli in a hue sequence as shown at the beginning of the test. Errors in the positioning of the different color disks were measured as indicator of the test performance [42]. Twenty-six workers were tested in the FM 100 test and the control group was composed by 26 subjects.

 Color discrimination ellipses were estimated using the Mollon-Reffin test for color discrimination evaluation [43]. The test had a pseudoisochromatic design, in which the target, a Landolt C, differed from the background only in chromaticity. Mean luminance of the target and background were the same. The target had 4.3° of outer diameter and 2.2° of inner diameter. The gap of the Landolt C was 1° of visual angle. The task of the subject was to identify the gap position. After each hit, the chromaticity of the target approached the chromaticity of the background. A staircase was used to estimate the minimum distance in chromaticity in the CIE1976 color space. Five background chromaticities were used (CIE1976 color space coordinates: E1. u′: 0.215, v′: 0.531; E2. u′: 0.219, v′: 0.481; E3. u′: 0.225, v′: 0.415; E4. u′: 0.175, v′: 0.485; E5. u′: 0.278, v′: 0.472), and each background chromaticity was discriminated from 8 chromaticity lines of different orientations. An ellipse fitted the threshold results. The area of a circle with equivalent area of the ellipses was chosen as indicator of color discrimination performance. Seventeen workers were tested in color discrimination ellipses and the control group was composed by 17 subjects.

### 2.2. Evaluation of the Exposure to Organic Solvents Mixture

Six out of 32 gas station workers reported use of individual safety instruments (masks and gloves). Mean duration of occupational exposure was 47.4 ± 61.7 months, with an exposure of 45.23 ± 4.4 hours/week.

### 2.3. Data Analysis

The normal range in each of the tests was defined by tolerance limits corresponding to 90% of the population with a 95% confidence [44]. The confidence interval was used to compare the exposed group with the control group. The *t*-test was used to compare data with one variable between gas station workers group and control group. Two-way ANOVA was used to compare the exposed group with the control group on data with more than one variable. Linear correlation was used to estimate the dependence of the psychophysical performance upon exposure time.

## 3. Results

### 3.1. Spatial Luminance Contrast Sensitivity

**Figure 1:**
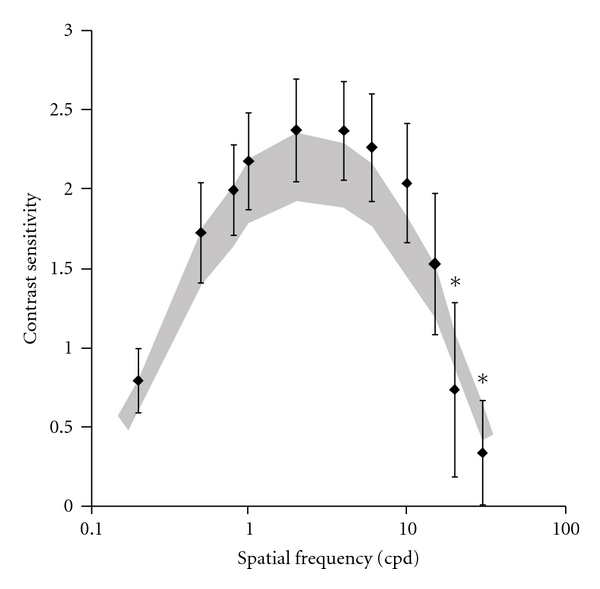
Mean spatial luminance contrast sensitivity at 11 spatial frequencies. Black diamonds represent the gas station workers contrast sensitivity and dark area represents the interval of confidence of control group.

Eight out of 25 gas station workers showed spatial luminance contrast sensitivity below the lower tolerance limit for at least one spatial frequency. Mean contrast sensitivity at 20 and 30 cpd of the gas station workers group was out of the interval of confidence of the mean of the control group (two-way ANOVA, *P* < 0.01; Figure 1). Correlations between the spatial luminance contrast sensitivity at different spatial frequencies and exposure time were very low (highest correlation (*r* ^2^) was lesser than 0.2).

### 3.2. Temporal Luminance Contrast Sensitivity

**Figure 2:**
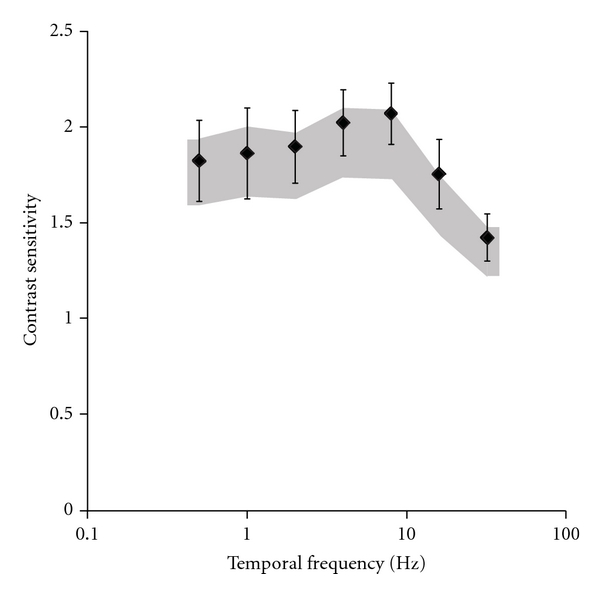
Mean temporal luminance contrast sensitivity at 7 temporal frequencies. Black diamonds represent the gas station workers contrast sensitivity and dark area represents the interval of confidence of control group.

All gas station workers showed temporal luminance contrast sensitivity within the control group tolerance limits. Mean contrast sensitivity was inside of the interval of confidence of the control (two-way ANOVA, *P* > 0.05; Figure 2). Correlations between the temporal luminance contrast sensitivity at different spatial frequencies and exposure time were very low (highest *r* ^2^ lesser than 0.1).

### 3.3. Visual Perimetry

**Figure 3:**
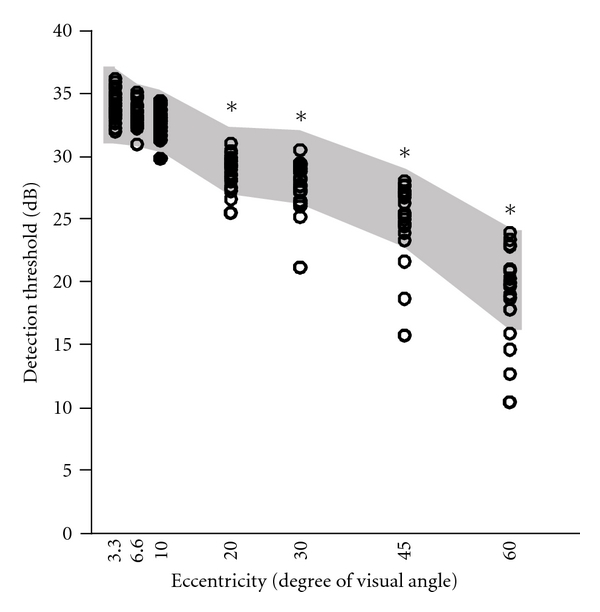
Detection threshold estimated by visual perimetry. Detection threshold as function of visual field eccentricity. Dark area represents the tolerance interval of detection thresholds of the control group.

Six out of 21 gas station workers had detection threshold below of the control tolerance limits for at least one eccentricity ring (Figure 3). Mean detection threshold of the exposed group was below the lower limit of confidence of control group in the rings of eccentricity between 10°–60°. Two-way ANOVA showed statistical difference of the detection threshold between both groups (*P* < 0.05). There was low linear correlation between the detection thresholds and exposure time (*P* < 0.45). Mean deviation (MD) and pattern standard deviation (PSD) of one worker was out of the control tolerance limits for the eccentricities below 10°. MD of four subjects and PSD of six subjects were out of the control tolerance limits for eccentricities between 10° and 30°. Two-way ANOVA showed statistical differences of MD values between both groups (*P* < 0.01) for eccentricities between 10°–30°, but no differences for MD values at eccentricities below 10° or PSD values for any eccentricity. Low linear correlations were found between MD or PSD and exposure time (*r* < 0.2).

### 3.4. Farnsworth-Munsell Hue 100 Test

**Figure 4:**
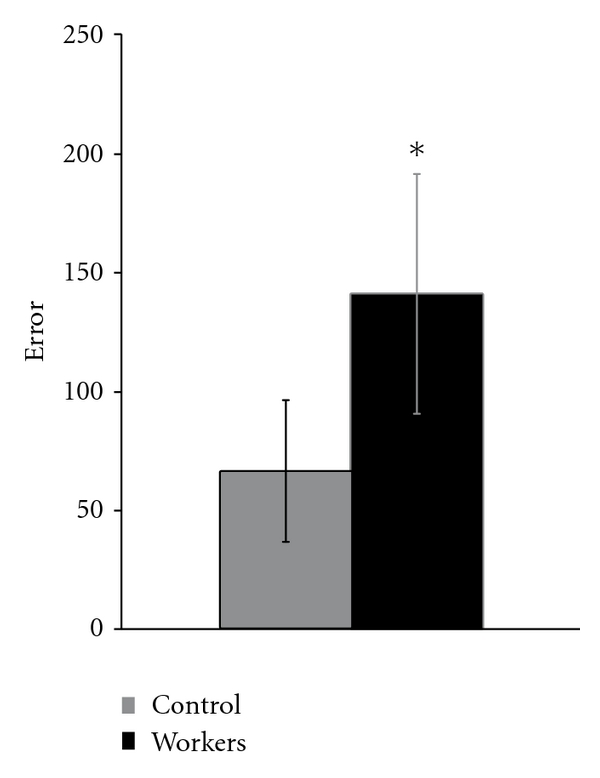
Mean FM100 errors of exposed (black bar) and control (gray bar) groups.

Fifteen out of 26 gas station workers had errors above the upper tolerance limit of the control group. Mean error value of the exposed group was higher than upper limit of confidence (*t*-test *P* < 0.01; Figure 4), low linear correlation between the exposure time and errors of FM100 test (*r* < 0.2).

### 3.5. Color Discrimination Ellipses

**Figure 5:**
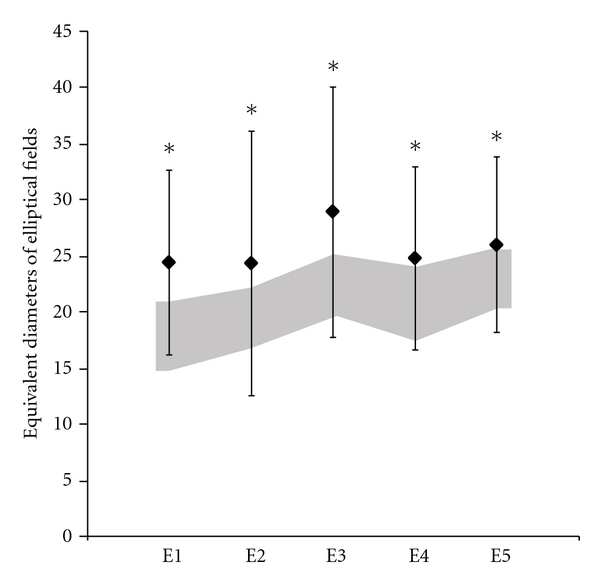
Equivalent circle diameter of color discrimination ellipses centered in five chromaticities in CIE1976. Black diamonds represent the equivalent circle diameter of elliptical area for different ellipses. Dark area represents the interval of confidence of the control group.

Six out of 17 workers showed increased equivalent circle diameter (*D*) to the area of the ellipse for at least one of five center references, when compared with the control tolerance limits. Mean *D* values of exposed group were higher than the upper limit of confidence of control group for all the color discrimination ellipses (Two-way ANOVA, *P* < 0.05; Figure 5). Low linear correlations were found between *D* from five ellipses and exposure time (*r* < 0.46).

## 4. Discussion

In the present study we assessed visual functions of gas station workers. In this profession, common in some countries, the job of the worker is to fill the automobile gas tanks. The worker is thus continuously exposed to a mixture of organic solvents throughout his work shift. We observed that twenty-five out of twenty-nine gas station workers had some kind of visual loss evaluated by psychophysical methods.

Many studies have demonstrated that workers exposed to organic solvent have visual impairments, mainly in color vision [3, 16–25, 45]. The mechanisms of neuronal dysfunction elicited by exposure to organic solvents are still unclear, but the affinity of organic solvent to lipid enriched tissues is well known. The nervous system is therefore a potential target of the solvent intoxication [46].

Most color vision studies have reported mainly blue-yellow color vision losses, and a secondary red-green color dyschromatopsia as shown in the present study [3, 16–25, 27–31]. Previous studies have investigated the color vision of solvent exposed workers using color arrangement test as FM100 test or Lanthony D15. As far we know, the present study is the first time that color discrimination ellipses test was applied in the solvent exposed subjects [16, 23–25, 27, 28, 30, 31, 38, 47–51]. As the tasks of color discrimination and FM100 test are quite different it is difficult to assert which test would be best to evaluate the color vision of the workers. This acquired dyschromatopsia might be the result of optics and neural causes [52, 53]. Aging can also lead to macular degeneration [52, 53]. The present study did not find worker diagnosed with any change in the ophthalmic clinical evaluation, suggesting that the color vision losses have neural predominant origin [31, 54].

Study in rats and nonhuman primates demonstrated an accumulation of metabolites from methanol in the vitreous and retina [55, 56], which could cause degeneration of outer nuclear layer and ganglion cell layer suggested for histopathologic studies by Potts and colleges [57]. For Köllner [58] blue-yellow color vision loss reflects changes in outer retina whilst losses in the red-green axes reflect abnormalities in the inner retina or optic nerve. This became known as Köllner's rule. Muttray et al. [22] argued that Köllner rule [58] could be combined with more recent findings [59], considering that outer retinal damage in the central retina would lead the subject to fixate at more eccentric retinal points. We agreed with Muttray's argument, because the pathologic eccentric fixation could result in impairment of red-green discrimination.

Dyschromatopsia associated to organic solvents intoxication has been attributed to maculopathies caused by damage in cone photoreceptors, ganglion cells and optic nerve demielinization [20, 32, 60]. Blain and Mergler [61] suggested that the fact solvent intoxication led to blue-yellow color vision losses and later may develop to red-green color vision loss, reflects progressive degeneration from outer retina to optic nerve [61]. Cortical changes in the visual processing can occur after organic solvent intoxication [16, 20, 32, 60, 62–64]. We described diffuse color vision losses, with no preferences for blue-yellow or red-green chromatic axes. This kind of color vision loss is associated to high exposure to organic solvents [16, 54].

Eight out of 25 gas station workers had luminance spatial contrast sensitivity lower than the tolerance limits defined by the control group. There was statistical difference between the organic solvent exposed workers and the control group at 20 and 30 cpd, but there was no change in their Snellen visual acuity. Boeckelmann and Pfister [6] and Järvinen and Hyvärinen [35] suggested measuring contrast sensitivity at low and intermediate spatial frequencies which reflect changes in the neural processing whereas loss of contrast sensitivity at high spatial frequencies reflects impairment of the optics of the eye. In the present work, all subjects had normal or corrected visual acuity to 20/20. Other studies on intoxication by organic solvents intoxication showed spatial vision impairments without changes of visual acuity [6, 21, 25, 35–40]. We found no impairments in the temporal contrast sensitivities in the organic solvent exposed workers.

Our results of visual field losses are similar to the findings of Yamamura [33]. Six out of 21 gas station workers had impairment of contrast sensitivity in eccentricities above 10°. Even the workers who were in the normal range of contrast sensitivity in eccentricities that ranged between 10°–60°, there was significant decreasing between the values of the exposed group and control group. This impairment is detected by MD (low values) and PSD (high values) analysis, reflecting in altered visual field with constriction of the visual field towards the central field. Grant and Schuman [34] suggest that this type of visual loss indicates a beginning process of optical neuropathy after exposure to methanol, styrene, toluene, trichloroethylene, and organic solvents mixtures.

In the present study, the exposed subjects have worked at the gas station from one month to twenty-one years (47.4 ± 61.7 months) and the period of exposure varied from 36 to 48 hours a week (45.23 ± 4.4 hours a week). Three subjects reported that they used protective safety equipment, but they lack specific training for use of this kind of equipment. Some studies found weak correlation between psychophysical performance of exposed subjects and their exposure to organic solvent mixtures, styrene, perchloroethylene, or benzene [22, 24, 65]. Although we also expected to find some correlation between total time of exposure and/or amount of daily exposure and the performance of exposed subjects in the psychophysical tests that we used, that was not the case. We did not find any significant correlation between the exposed subject performances and the duration or amount of exposure to organic solvents.

Concentration of organic solvents or their metabolites in tissues are not directly related to time of exposure. There are genes that code enzymes that work in the metabolism of organic solvents in the organisms, and gene polymorphism modifies the absorption and the neurotoxicity effects of the organic solvents [25, 66]. We suggest that visual system damage probably occurred at early moment of solvent exposure, and the intersubject variability in the psychophysical tests could be explained by individual genetic predisposition.

The current study investigated psychophysically the achromatic and chromatic vision of gas station workers, and correlated the psychophysical results with time of exposure. These results have previously been published as abstracts [67].
